# Preparation and Characterization of Guar-Montmorillonite Nanocomposites

**DOI:** 10.3390/ma6115199

**Published:** 2013-11-13

**Authors:** Rola Mansa, Christian Detellier

**Affiliations:** Centre for Catalysis Research and Innovation and Department of Chemistry, University of Ottawa, 10 Marie Curie, Ottawa, Ontario K1N 6N5, Canada; E-Mail: rmansa@uottawa.ca

**Keywords:** exfoliation, guar, intercalation, montmorillonite, nanocomposite, polymer

## Abstract

Polymer-clay nanocomposites are highly sought-after materials, mainly due to their applicability in a variety of avenues. From the standpoint of the preparation of these nanocomposites, however, organic compatibility with clay and adherence to “green chemistry” concepts and principles can be limiting factors. As such, the objective was to prepare a biopolymer-modified clay nanocomposite using a simple and environmentally friendly method of preparation, whereby pre-treatment of the clay for organic compatibility was bypassed. Novel montmorillonite nanocomposites were prepared using neutral guar gum and cationic guar gum. X-ray diffraction (XRD) and transmission electron microscopy (TEM) confirmed the formation of intercalated structures. A monolayer configuration of cationic guar within the interlayer space was indicated by XRD results, while treatment with neutral guar gum resulted in the observance of both monolayer and bilayer configurations. Additionally, TEM results indicated partial exfoliation. Results attributed from ^13^C cross polarization/magic angle spinning nuclear magnetic resonance spectroscopy (CP/MAS NMR) of the nanocomposites indicated peaks corresponding to the guar constituent, confirming the adsorption of the biopolymer. Inductively coupled plasma emission spectrometry (ICP-ES) results indicated the exchange of cations present in neutral guar gum with the sodium cations of montmorillonite, in the case of the neutral guar nanocomposites.

## 1. Introduction

Polymer layered silicates (PLS), an intensely researched class of nanocomposites, are the products of the association of polymers and layered silicates, forming a continuous phase containing a dispersed phase, with at least one dimension at the nanometer scale [1,2,3,4]. Great interest has been generated in the use of PLS nanocomposites due to a synergism of properties inherent to both the organic and inorganic constituents [5,6,7], with a potentiality for applications in many fields, including drug delivery systems [8,9,10,11,12,13]. Clay minerals are often used in conventional pharmaceutical applications as excipients and active agents [14]. The clay mineral montmorillonite (MMT), a layered aluminosilicate from the smectite family, has a cationic exchange capacity, as well as adsorption properties, and as such has received considerable attention towards its use for pharmaceutical applications [14,15]. As an excipient, montmorillonite is usually modified to improve its affinity for drug molecules and one such mode of modification involves interaction with a polymer [15].

Several examples of montmorillonite modified with polymers, for the purposes of drug delivery, are described in the literature [9,12,13,15,16,17]. Among the advantages associated with such a delivery system is the ability to modulate drug release [9,13,16,18]. For example, the controlled release of dexamethasone was investigated using nanocomposites based on poly (ethylene-vinyl acetate) and a pre-treated montmorillonite [16]. As well, *N-*isopropylacrylamide-montmorillonite nanocomposite hydrogels were synthesized and investigated in terms of physical properties and drug release behavior [17].

The modification of clay minerals by intercalation of biopolymers pertains to the current trend of the development of green chemistry. The impetus towards natural polysaccharide-modified clay is based on the general biodegradability, environmental friendliness and renewable nature of these biopolymers [19,20], which is in accordance with the concept of green nanocomposites [21]. Moreover, biopolymers are abundant in nature and available at a low cost [19]. Among the polysaccharides investigated for the modification of montmorillonite, much interest had been generated in chitosan, including the aspect of control on whether the chitosan intercalated as a monolayer or a bilayer [3,22,23,24,25]. Cellulose was also used to form polymer-clay nanocomposites with montmorillonite [26].

**Figure 1:**
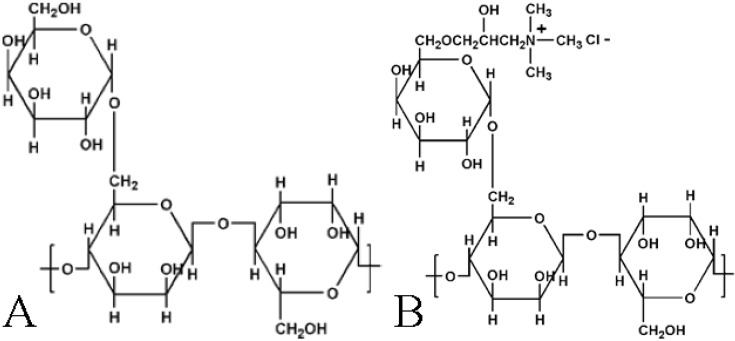
Structure of (**A**) Neutral guar gum (NG); and (**B**) Cationic guar gum (CG).

Guar Gum is a galactomannan polysaccharide obtained from the endosperm of the seed of *Cyamopsis tetragonoloba* [27,28]. This gum consists of a backbone of (1–4) β-D-manno-pyranosyl units with every second unit bearing an (1–6) α-D-galacto-pyranosyl unit (Figure 1A). Guar Gum is a high molecular weight polysaccharide with a previous study indicating the molecular weight as between 800 and 5000 kDa [29]. Guar hydroxypropyl trimethyl ammonium chloride (Figure 1B) is a quaternary ammonium derivative of guar gum [27]. Guar gum and the modified forms are predominantly used in the food industry [30].

Guar gum is a good candidate to modify montmorillonite for potential pharmaceutical applications. Its intercalation would increase the interlayer space of montmorillonite which can then potentially effectuate the further intercalation of other organic molecules [31] for example, drug molecules for pharmaceutically interesting applications. Along with increasing the interlayer space, the intercalated guar gum contains many hydroxyl groups, which may allow interactions to occur with drug molecules, also potentially encouraging the uptake of drug compounds by montmorillonite.

Although the flocculation of bentonite suspensions using cationic guar derivatives were previously studied [27], the present article reports the first preparation and characterization of guar-montmorillonite nanocomposites prepared by the solvent intercalation method, using montmorillonite purified according to conventional methods [32,33], and neutral and cationic guar gum. Characterization was performed by X-ray diffraction (XRD), transmission electron microscopy (TEM), thermogravimetric analysis (TGA), solid-state ^13^C cross polarization/magic angle spinning nuclear magnetic resonance (^13^C CP/MAS NMR), solid-state ^23^Na magic angle spinning nuclear magnetic resonance (^23^Na MAS NMR) spectroscopy, and inductively coupled plasma emission spectrometry (ICP-ES) were used for characterization purposes

## 2. Results and Discussion

### 2.1. Interlayer Structure of the Modified Montmorillonite

**Figure 2:**
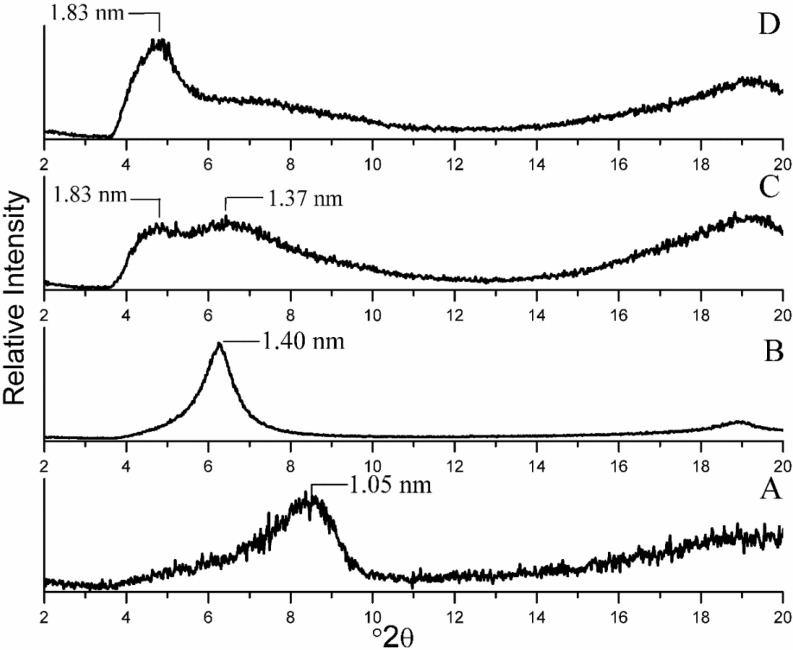
X-ray diffraction (XRD) patterns of (**A**) Na^+^ mineral montmorillonite (MMT); (**B**) CG-MMT 3:1; (**C**) NG-MMT 3:1; (**D**) NG-MMT 6:1.

The XRD patterns of the nanocomposites were all obtained using dried XRD mounts. The predominant absence of water in the interlayer space is indicated by the d_001_ peak with a maximum at 1.05 nm for Na^+^ MMT, which was near the range designated for dehydrated montmorillonite [34]. Comparison of the XRD patterns of starting Na^+^ MMT (Figure 2A) and CG-MMT 3:1 (Figure 2B) revealed the enhancement of the d_001_ value from 1.05 nm to 1.40 nm, which indicated an intercalation of cationic guar gum. The orientation of the intercalated polysaccharide molecules was deduced from the basal spacing observed. When the 0.95 nm thickness of the silicate layer [35] was subtracted, a resultant 0.45 nm interlayer spacing was calculated, which was in accordance with a monolayer orientation of a polysaccharide, as based on a representative 0.38 nm thickness of a sheet of polysaccharide chains, derived from the XRD diffraction pattern of a comparable polysaccharide, chitosan [22,23,24]. The XRD patterns of NG-MMT 3:1 (Figure 2C) and NG-MMT 6:1 (Figure 2D), as compared to the starting Na^+^ MMT (Figure 2A) also revealed an enhancement of the d_001_ values, which indicated the intercalation of neutral guar gum within the montmorillonite layers. In the case of NG-MT 3:1, a broad feature split into two was apparent and this feature was interpreted as the d_001_ reflection owing to the intercalation of guar gum in an intermediate state with the existence of both monolayer and bilayer orientations. The interlayer spacing of 0.42 nm and 0.88 nm corresponded approximately to a monolayer orientation and a bilayer orientation respectively, as deduced by subtraction of the thickness of the silicate layer from the inferred d_001 _values of 1.37 and 1.83 nm [24]. In the case of NG-MMT 6:1, a diffraction feature with a d_001_ maximum centered at 1.83 nm was present, and this indicated that the bilayer orientation intercalated more predominantly. In the case of the neutral guar nanocomposites, an evident increase in broadness of the d_001_ reflection, as compared to that of CG-MMT 3:1 and Na^+^ MMT, was observed, suggesting a degree of disorder and irregularity involved in the expansion of the clay layer during the intercalation of neutral guar gum [1].

The intensity scales of the XRD traces of the nanocomposites have been adjusted to allow for identification of the d_001_ diffraction*.* In the case of the neutral guar nanocomposites (Figure 2 C,D), the traces are characterized by greater-intensity backgrounds and lower-intensity d_001_ diffraction peaks, as compared to CG-MMT 3:1. Along with the d_001_ peak shift towards lower 2θ angles, the presence of these features in the XRD trace of a composite may indicate the concurrent presence of organoclay layers that are in a disordered, exfoliated and intercalated state, where the clay layers are well-dispersed in the polymer matrix [36]. On the other hand, the CG-MMT 3:1 nanocomposite (Figure 2B) is characterized by a lower-intensity background and high intensity d_001_ diffraction peak shifted towards a lower 2θ angle, indicating the existence of a well-ordered, intercalated structure. This suggests that an electrostatic interaction of the cationic guar gum with the negatively charged clay layers may have affected the extent of the ordering of the clay layers within the polymer matrix.

### 2.2. Morphology of the Nanocomposites by TEM Imaging

**Figure 3:**
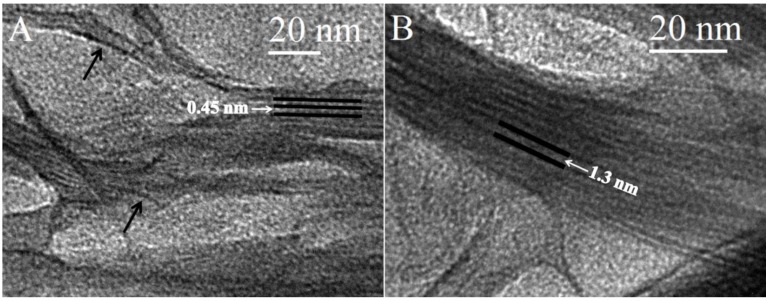
Transmission electron microscopy (TEM) images of CG-MMT 3:1.

TEM imaging was used to elucidate the morphology of the nanocomposites. Staining of the samples was unrequired due to the high electron density difference between clay and polymer [37]. The images acquired of CG-MMT 3:1 (Figure 3) displayed stacks with an average length of 10–25 nm. The length of the stacks of the nanocomposite were smaller than those observed in the images of Na^+^ MMT (Figure 6), indicating that the clay layers dispersed into smaller stacks in the polymeric matrix of cationic guar gum [38]*.* Curvature of the clay platelets, noted in literature with other nanocomposites, was also observed in the images of CG-MMT 3:1 (black arrows on Figure 3A) [39,40]. In Figure 3A, the clay layers of the stack appeared as dark lines bordering interlayer spaces that measured an average of 0.45 nm, in agreement with the XRD results which depicted an intercalated structure with the same average interlayer space. While the peak maximum of the 001 reflection for CG-MMT 3:1 represented an interlayer space of 0.45 nm, Figure 3B displayed a stack of clay layers where an interlayer space of approximately 1.3 nm was measured. This greater expansion of the interlayer space remained in agreement with XRD results, considering that the peak that corresponded with the 001 reflection of CG-MMT 3:1 was broad and encompassed a range of d-spacing values, including 1.3 nm. Thus, the TEM results were in good agreement with XRD results; nonetheless, as reported previously by another study, an XRD-acquired value for the interlayer spacing of a nanocomposite composed of intercalated and exfoliated regions does not represent a discrete, actual value for the expansion of the clay layers, but was an average value of the mixed morphologies [38].

**Figure 4:**
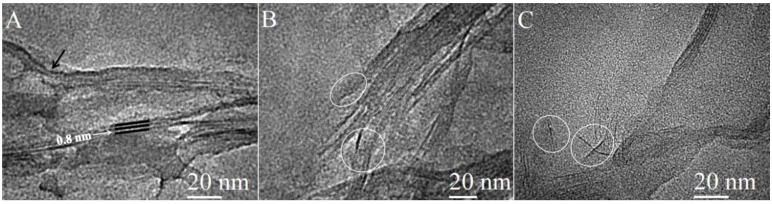
TEM images of NG-MMT 3:1.

TEM images of NG-MMT 3:1 (Figure 4) indicated clay layers aligned in a parallel manner and stacked together in domains with varying sizes, ranging from approximately 5 nm to 40 nm, validating the intercalation effect inferred from XRD results. The interlayer space was measured from several of the images obtained and was found to be in the range of 0.45–0.85 nm. A clay stack with interlayer space of 0.8 nm can be observed in Figure 4A. The measured range of values of the expansion of the interlayer space was as expected, based on the intermediacy of the structure of NG-MMT 3:1, depicted by the XRD results, and the nature of XRD-derived results in presenting average values of the d-spacing. Curvature of the clay layers was also observed in the case of NG-MMT 3:1 (black arrow on Figure 4A). Smaller stacks of clay than those present in Na^+^ MMT are observed in NG-MMT 3:1, an example of which was observed in Figure 4A, where a stack consisted of only three clay layers, in contrast to the larger stack observed in the image of Na^+^ MMT (black arrow on Figure 6A). Moreover, detachment of the clay layers in two regions are observed, whereby a smaller stack of clay appeared to be in the early stages of breaking off or segregating from the parent stack of clay (circled on Figure 4B), evidence of the concurrent exfoliation effect present along with the intercalated morphology. Upon close inspection of Figure 4C, the presence of single clay mineral layers was observed (circled), indicating the individual dispersion of the exfoliated sheets in the polymer matrix [38,39].

**Figure 5:**
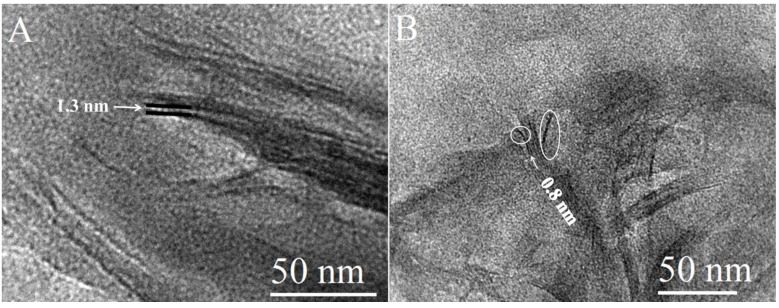
TEM images of NG-MMT 6:1.

The images of NG-MMT 6:1 (Figure 5) also depicted those of a nanocomposite composed of small stacks of intercalated clay layers, in this case, in domains ranging from 10 nm to 30 nm. The interlayer spacing ranged from 0.8 nm to 1.3 nm, in agreement with XRD results. Notably, NG-MMT 6:1 displayed the detachment of an individual clay layer from a smaller stack (circled). Interestingly enough, the separation of stacks was observed in the case of NG-MMT 3:1 (Figure 4B), and when the concentration of polymer was increased, as in the case of NG-MMT 6:1, the detachment of single clay layers from a stack was observed (Figure 5B).

**Figure 6:**
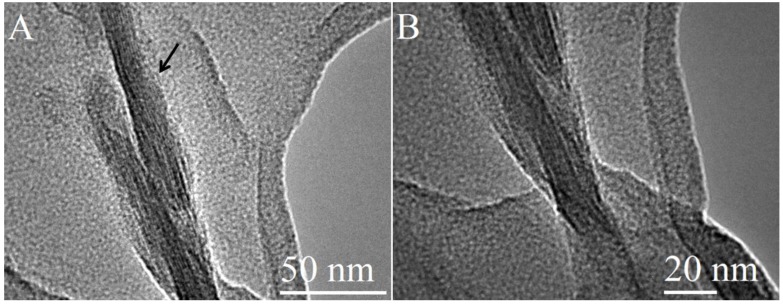
TEM images of Na^+^ MMT.

### 2.3. Thermal Analysis

**Figure 7:**
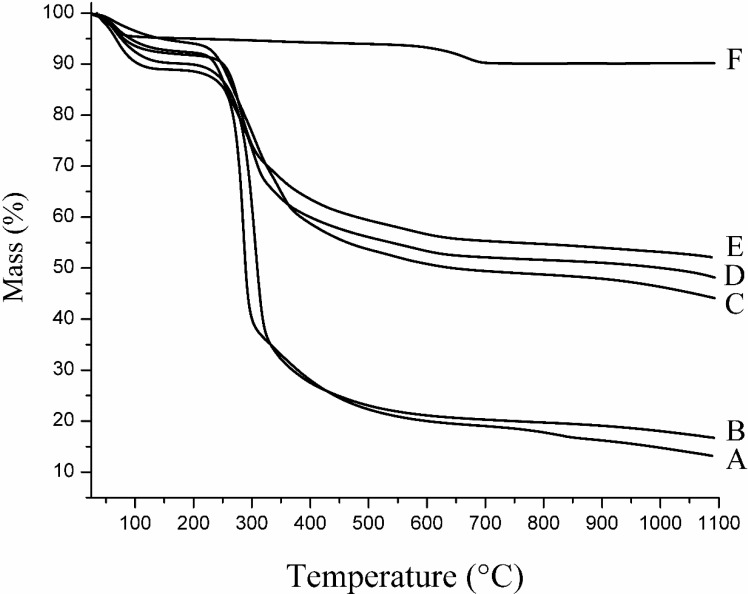
TG traces of (**A**) CG; (**B**) NG; (**C**) NG-MMT 6:1; (**D**) NG-MMT 3:1; (**E**) CG-MMT 3:1; and (**F**) Na^+^ MMT.

**Figure 8:**
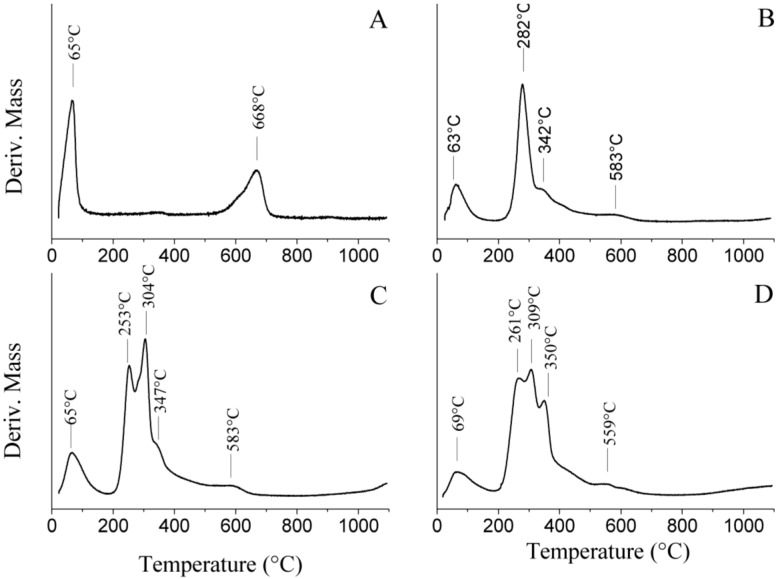
Differential thermal gravimetric (DTG) traces of (**A**) Na^+^ MMT; (**B**) CG-MMT 3:1; (**C**) NG-MMT 3:1; and (**D**) NG-MMT 6:1.

Thermogravimetric analysis of Na^+^ MMT revealed two distinct mass loss steps (Figure 7F). First, the montmorillonite underwent dehydration of the adsorbed water within the temperature range of 25–100 °C, corresponding to a mass loss of approximately 5%. This stage was followed by dehydroxylation occurring within the temperature range of 560–700 °C observed on the TG trace [41,42]. In the case of the nanocomposite CG-MMT 3:1, the initial loss of adsorbed water was evidenced by a mass loss step starting at 50 °C (Figure 7E), and a corresponding broad differential thermal gravimetric (DTG) peak centered at 63 °C (Figure 8B). The TG trace of CG-MMT 3:1 indicated the pyrolysis of the guar constituent occurring within the temperature range of 220–500 °C (Figure 7E). The total mass loss of CG-MMT 3:1 (Figure 7E) was greater than that of Na^+^ MMT (Figure 7F) by 38.0%, confirming the uptake of the cationic guar gum. Moreover, the temperature range of this mass loss correlated with the temperature range of the mass loss seen on the TG trace of cationic guar gum (CG) (Figure 7A). The DTG trace of CG revealed the decomposition as a single sharp peak centered at 287 °C (not shown). In the case of the nanocomposite, the peak that corresponded to the pyrolysis of the cationic guar gum constituent was broad with a shoulder at 342 °C (Figure 8B), and this difference was interpreted as the effect of polymer interaction with the clay.

Likewise, the neutral guar nanocomposites NG-MMT 3:1 (Figure 7D) and NG-MMT 6:1 (Figure 7C) showed total mass losses of 51.7% and 55.8%, respectively; these mass losses exceeded the total mass loss associated with the TGA of Na^+^ MMT (Figure 7F), and this confirmed the adsorption of the neutral guar gum. Moreover, the pyrolysis of the guar constituent of the nanocomposites occurred within a temperature range which corresponded to pyrolysis of the neutral guar gum (NG) (Figure 7B). The DTG trace of NG-MMT 3:1 (Figure 8C) indicated the loss of neutral guar by two peaks centered at 253 °C and 304 °C, and a shoulder centered at approximately 347 °C. The DTG trace of NG-MMT 6:1 (Figure 8D) indicated the loss of neutral guar as three peaks centered at 261 °C, 309 °C, and 350 °C. This differed from the single sharp DTG peak centered at 305 °C associated with the pyrolysis of solely the neutral guar gum (not shown). If the loss of neutral guar gum was analogous to that of alkylammonium ions, then the loss of the organic component in multiple steps would have been indicative of differing environments, and thus differing thermal stabilities [43]. In that case, the presence of several peaks corresponding to the loss of the neutral guar gum was indicative of its external adsorption onto the clay surface, along with its intercalation, with the predominant loss of the externally adsorbed guar gum occurring within the mass loss step associated with the DTG peak at 253 °C, in the case of NG-MMT 3:1, and at 261 °C, in the case of NG-MMT 6:1 (Figure 8C,D). Likewise, if the thermal stability of the organic compound was influenced by its interaction with clay, the pyrolysis of externally adsorbed cationic guar gum likely occurred within the temperature range associated with the DTG peak at 282 °C (Figure 8B).

The temperature of the dehydroxylation of Na^+^ MMT, with a DTG peak centered at 668 °C was higher than that of CG-MMT 3:1 (583 °C), NG-MMT 3:1 (583 °C ), and NG-MMT 6:1 (559 °C) (Figure 8). In many cases, the thermal analysis of organo-clay complexes reveals a decrease in the temperature of dehydroxylation of the clay components, usually by a difference of 30–90 °C, and this was reported for both kaolin clays and smectites [44,45,46,47]. The interaction of the guar gum with the hydroxyl groups of montmorillonite may have provided a mechanism to induce the early onset of dehydroxylation [46,47].

Although the adsorption process is usually associated with a concurrent desorption of water molecules [48], the TG trace of the nanocomposites revealed a greater content of physically adsorbed water, in comparison to starting montmorillonite. Guar gum is hygroscopic in nature [49], and so this oddity was interpreted as a result of hygroscopic guar gum that remained surface adsorbed despite washing and centrifugation.

### 2.4. Structural Characterization

**Figure 9:**
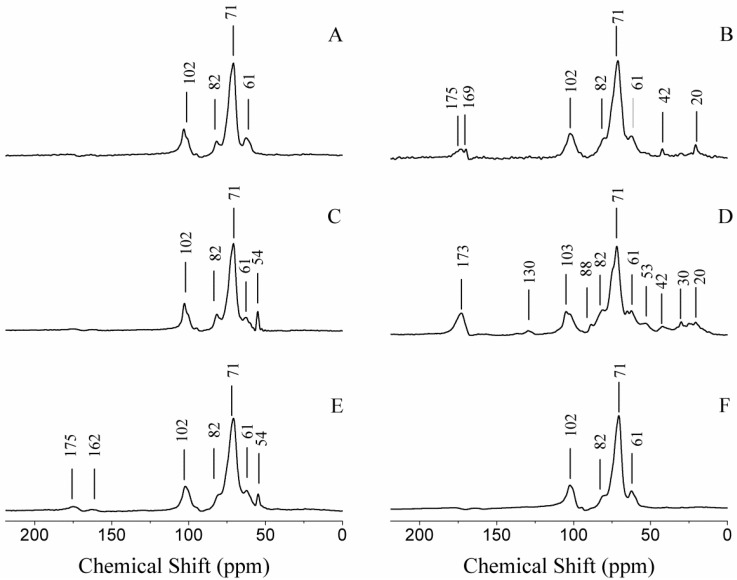
^13^C CP/MAS NMR spectra of (**A**) NG; (**B**) NG-MMT 3:1; (**C**) CG; (**D**) NG-MMT 6:1; (**E**) CG-MMT 3:1; and (**F**) NG Control.

For the purpose of the characterization of the nanocomposites, ^13^C CP/MAS NMR was used to show the presence of the polymer and to ensure its structural integrity and ^23^Na MAS NMR was used to monitor the exchange of the interlayer sodium cations with other cations, in this case cationic guar gum. Indeed, the ^13^C CP/MAS NMR spectrum of CG-MMT 3:1 (Figure 9E) displayed signals that corresponded to cationic guar gum (Figure 9C), indicating the presence of the polymer. The ^13^C CP/MAS NMR spectrum of NG-MMT 3:1 (Figure 9B) and NG-MMT 6:1 (Figure 9D) also revealed signals that corresponded to neutral guar gum (Figure 9A), as well as the presence of other unexpected peaks, minor ones, seen in the range of 20–53 ppm. More prominently, peaks at 169 ppm and 175 ppm, which were seen in the ^13^C CP/MAS NMR spectrum of NG-MMT 3:1 (Figure 9B), and a broad peak centered at 173.0 ppm, which was seen in the ^13^C CP/MAS NMR spectrum of NG-MMT 6:1 (Figure 9D)*,* indicated the existence of carboxylic acid carbons, absent in starting guar gum (Figure 9A). A control experiment was performed with neutral guar gum (NG Control), in order to examine the causative factor for the presence of carboxylic acids. The ^13^C CP/MAS NMR spectrum of NG Control (Figure 9F) revealed an absence of peaks in the 170.0–175.0 ppm region, which was an indication that montmorillonite did indeed play a role in the presence of carboxylic acid carbons in the NMR spectra of the nanocomposites. Literature indicated that carboxyl functional groups on guar gum molecules did exist in small amounts in commercial guar gum [29], which led to the interpretation that the presence of carboxylic acid carbons on the NMR spectra and their absence in the spectrum of NG Control was possibly due to the preferential accumulation by montmorillonite of guar polymers containing carboxyl functional groups, along with other groups that were evidenced as minor peaks in the range of 20–53 ppm. Another interpretation involved montmorillonite, a known catalyst for a number of organic reactions, playing a catalytic role [50]. Based on a literature search performed, however, any evidence of montmorillonite reacting with polysaccharides to catalyze the formation of carboxyl groups has yet to be reported.

**Figure 10:**
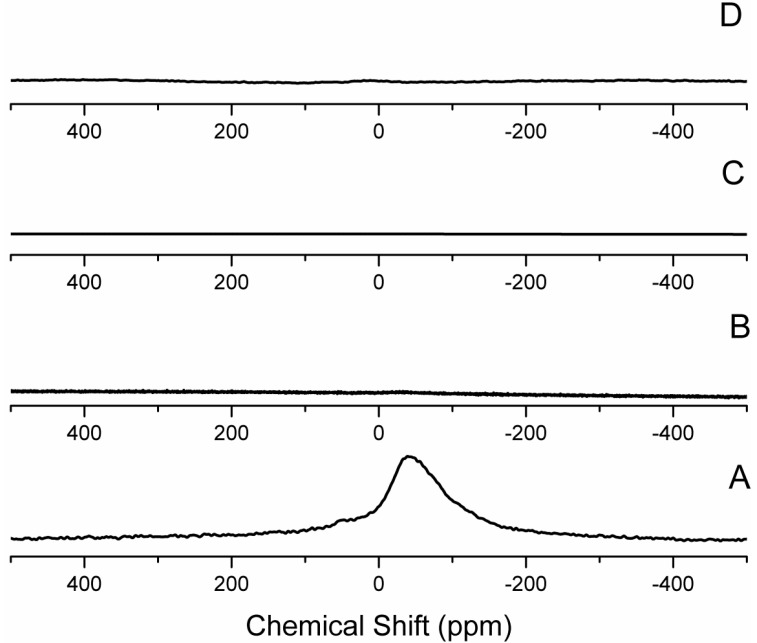
^23^Na MAS NMR spectra of (**A**) Na^+^ MMT; (**B**) CG-MMT 3:1; (**C**) NG-MMT 3:1; and (**D**) NG-MMT 6:1.

The ^23^Na MAS NMR spectrum of CG-MMT 3:1 (Figure 10B) revealed the absence of sodium cations in the interlayer space. The intercalation of cationic compounds occurs through the conventional cation exchange method, whereby the sodium cations in the interlayer space of Na^+^ MMT are exchanged for cationic guest molecules. Consequently, the absence of sodium cations is expected. When neutral polymers are intercalated, co-existence of the polymer and of the original cations is expected [51]. The ^23^Na MAS NMR spectra of NG-MMT 3:1 (Figure 10C) and NG-MMT 6:1 (Figure 10D), however, indicated an absence of the sodium cations. Literature indicated the possible association of potassium, calcium, zinc, sodium, and iron, with guar gum [52]. The presence of these cations was likely to be the cause of the absence of sodium cations in the nanocomposite and a chemical analysis was performed to confirm their presence.

### 2.5. Chemical Analysis

The chemical analysis through ICP-ES of Na^+^ MMT (Table 1) indicated a 1.65% value for Na, which was highest in concentration when compared to the other cations that are not structural constituents of montmorillonite. This indicated that the initial sodium-saturation step was efficiently accomplished. The measured concentrations of the structural elements of the nanocomposites, which included Al, Mg, Si, and Fe, were lower than those observed in Na^+^ MMT (Table 1). When taking into account the clay content of the nanocomposites, the measured concentrations of the structural elements did not vary significantly from their expected structural concentrations. The concentrations of the structural elements of CG-MMT were 45%–54% of those present in Na^+^ MMT. As for NG-MMT 3:1, the concentrations of the structural elements present were 40%–52% of those present in Na^+^ MMT. In the case of NG-MMT 6:1, the concentrations of the structural elements were 32%–45% of those present in Na^+^ MMT. Overall, this matched the pattern of decreasing clay content with increasing guar gum content, previously established by TGA.

While the quantities of the structural elements of Al, Fe, Mg, and Si, in the nanocomposites were in close agreement with their expected structural contributions, the quantity of Na, the exchangeable cation, did indeed vary as compared to that of starting Na^+^ MMT. A decrease in the content of Na was expected and was indeed observed in the case of CG-MMT 3:1 (Table 1). The lower concentration of Na in CG-MMT 3:1 (0.082%) as compared to Na^+^ MMT (1.65%), indicated that the intercalation had occurred through a conventional cationic exchange mechanism (Table 1). On the other hand, the concentration of Na in NG-MMT 3:1 (0.431%) and NG-MMT 6:1 (0.305%) was lower than expected, given that an intercalation of solely the neutral variant of guar gum should not have affected the concentration of sodium cations in the interlayer space (Table 1). The low values of Na present in the neutral guar nanocomposites indicated that an exchange had occurred in the reaction media involving the sodium cations of the starting montmorillonite. On the other hand, the concentrations of K and Ca in the neutral guar nanocomposites had increased in comparison to those present in the starting Na^+^ MMT (Table 1). ICP-ES analysis was performed on neutral guar gum and cationic guar gum, and the results verified that the biopolymers were the source of these extraneous metal ions (Table 2). In the case of the neutral guar nanocomposites, this signified the occurrence of an exchange process between cations associated with the neutral guar gum and the sodium cations of the starting Na^+^ MMT, along with the intercalation of the neutral guar gum, hence the decreased concentration of sodium and the lack of sodium signal observed through NMR.

**Table 1:** Elemental Content Measured in starting Na^+^ mineral montmorillonite (MMT) and the nanocomposites.

Element	Na^+^ MMT	CG-MMT 3:1	NG-MMT 3:1	NG-MMT 6:1
Al (wt %)	9.666	5.067	4.527	3.638
Ca (wt %)	0.047	0.075	0.119	0.162
Fe (wt %)	2.614	1.308	1.222	0.987
K (wt %)	0.110	0.063	0.250	0.273
Mg (wt %)	1.199	0.645	0.622	0.535
Na (wt %)	1.649	0.082	0.431	0.305
Si (wt %)	28.69	12.84	11.47	9.25
Cu wt (μg/g)	6.3	9.3	19.1	12.7
Mn wt (μg/g)	90.4	44.3	42.6	39.8
Zn wt (μg/g)	92.0	61.3	68.9	60.0

**Table 2:** Elemental Content Measured in neutral guar gum (NG) and Cationic guar gum (CG).

Element wt (μg/g)	CG	NG
Al	1.85	3.56
Ca	381.0	518.2
Fe	19.5	28.8
K	1357	1886
Mg	234	293
Na	4732	147
Si	4.62	10.94
Cu	1.31	1.14
Mn	1.261	2.025
Zn	3.95	4.52

## 3. Experimental Section 

### 3.1. Materials

Cationic Guar Gum (CG) was purchased from Spec-Chem Industry (Nanjing, China) and used as received. Neutral Guar Gum (NG) was purchased from Sigma-Aldrich (St Louis, MO, USA) and used as received. Montmorillonite SWy-2 (MMT) was obtained from the Source Clays Repository of The Clay Minerals Society (West Lafayette, IN, USA). Purification was performed by the conventional method as follows: MMT was suspended in deionized water and the <2 μm fraction was collected and then sodium-saturated by dispersion in 1 M NaCl solution. The clay mineral was then washed in deionized water followed by centrifugation for recuperation. This process was repeated four times after which the clay was dialyzed in dialysis bags to remove the excess salt [32,33].

### 3.2. Preparation of Guar-Montmorillonite Nanocomposites (CG-MMT 3:1, NG-MMT 3:1, NG-MMT 6:1)

The preparation of CG-MMT 3:1 involved first the addition of 9.0 g of cationic guar gum (CG) to 1.5 L of water, followed by the addition of 3.0 g of starting montmorillonite (Na^+^ MMT), while vigorously stirring. The resultant dispersion was stirred at room temperature for three weeks. The product was recovered through four cycles of washing/centrifuging using water, dried at 50 °C for 4 h, and then ground into a powder using a mortar and pestle. The preparation of NG-MMT 3:1 involved first the addition of 7.5 g of neutral guar gum (NG) to 1.5 L of water, followed by the addition of 2.5 g of Na^+^ MMT, while vigorously stirring. To prepare NG-MMT 6:1, 15 g of neutral guar gum were added to 1.5 L of water, followed by the addition of 2.5 g of Na^+^ MMT, also while vigorously stirring. Both dispersions were stirred at room temperature for three weeks, and both products were recovered and handled in the same way as CG-MMT 3:1.

### 3.3. Preparation of NG Control

A control experiment was performed where 15 g of neutral guar was added to 1.5 L of water and stirred at room temperature for three weeks. The product was recovered and handled in the same way as CG-MMT 3:1.

### 3.4. Characterization

X-ray diffraction patterns (XRD) were obtained on a Philips PW 3710 diffractometer, with a Cu Kα radiation at a wavelength of 0.154 nm, a generator voltage of 45 kV and current of 40 mA. XRD mounts for each sample were prepared by pipetting a small amount of water-dispersed sample onto an XRD slide and drying in the oven at approximately 50 °C for 40 min. To ensure the comparability of the XRD measurements of the various samples, the drying of the XRD mounts was performed at the same temperature and time duration for all of the samples, and the XRD measurements were taken shortly thereafter. Thermogravimetric analysis (TGA) data were recorded using an SDT 2960 instrument under N_2_ flow (120 mL/min) with a heating rate of 10 °C/min. Solid-state ^13^C cross polarization/magic angle spinning nuclear magnetic resonance (^13^C CP/MAS NMR) and ^23^Na magic angle spinning nuclear magnetic resonance (^23^Na MAS NMR) spectra were collected using a Bruker AVANCE 200 NMR spectrometer. The ^13^C spectra were acquired at 50.31 MHz with contact time of 1200 μs and relaxation delay of 1 s. The ^13^C CP/MAS NMR signals were externally referenced to the signal of glycine at 176.03 ppm. The ^23^Na MAS NMR spectra were acquired at 52.93 MHz with a relaxation delay of 1 s. The ^23^Na MAS NMR signals were externally referenced to the signal of sodium chloride at 7.21 ppm. Typical spinning rates for ^13^C NMR experiments and ^23^Na NMR experiments were 4.5 kHz. For the purpose of preparing the sample for transmission electron microscopy (TEM) images, powdered samples were embedded into LR White resin. Thin samples were then sectioned by a Leica EM UC6 microtome (Leica microsystems, Wetzlar, Germany), equipped with a diamond knife (Diatome, Hatfield, PA, USA). Sections were transferred to copper TEM grids (300 mesh EMS), and coated with carbon. Images were acquired using a JEOL JEM-2100F field emission transmission electron microscope equipped with an ultra high resolution pole-piece operating at 200 kV. Inductively coupled plasma emission spectrometry (ICP-ES) was performed using an Agilent VistaPro CCD ICP-ES Spectrometer. Nanocomposite samples and a standard reference material NIST 2709 (San Joaquin Soil) from NIST (National Institute of Standards and Technology, Gaithersburg, MD, USA) were sequentially digested in aqua regia, followed by HF-HNO_3_. The validity of the digestion method was ensured by the close agreement of the results of the standard reference material NIST 2709 (San Joaquin Soil) with certified values [53]. Na^+^ MMT and SRM NIST 2709 (San Joaquin Soil) samples were analyzed in quadruplicate and quintuplicate portions, respectively, of approximately 100 mg to evaluate the overall method precision, including sample homogeneity. A single portion of CG-MMT 3:1 was analyzed, and duplicate and quadruplicate portions of NG-MMT 3:1 and NG-MNMT 6:1 were analyzed, respectively. Sample preparation for the neutral guar gum and cationic guar gum were performed by dry ashing, followed by dissolution in aqua regia. Triplicate portions of the gums were analyzed.

## 4. Conclusions

Novel guar-montmorillonite nanocomposites were prepared by the solution intercalation method. While analysis of their XRD patterns indicated intercalation resulting from treatment of montmorillonite by guar gum, TEM provided visible evidence of said intercalation as well as the presence of a partial exfoliation effect. A monolayer configuration of the cationic guar in the interlayer space was observed while treatment with neutral guar gum provided an expansion of the interlayer space that was equated with the presence of both monolayer and bilayer configurations. At a higher neutral guar gum content, a predominant formation of the bilayer was present, which indicated the ability to control the configuration of the nanocomposite. The intercalation of guar gum was accompanied by its external adsorption, as well as by the exchange of the montmorillonite sodium cations by cationic species associated with guar.

The observed morphology and structure of the guar-montmorillonite nanocomposites show a dependence on the relative amounts of guar and montmorillonite used for their preparation. This will provide the necessary flexibility for the potential applications of these new nanocomposites.
